# Non-invasive multimodal spinal network stimulation instantaneously recovers locomotor function after paralysis

**DOI:** 10.1093/nsr/nwaf239

**Published:** 2025-07-08

**Authors:** Yury Gerasimenko, Susan Harkema

**Affiliations:** Pavlov Institute of Physiology, Russia; Department of Physiology, University of Louisville, USA; Tim and Caroline Reynolds Center for Spinal Stimulation, Kessler Foundation, USA

Historically, individuals with clinically complete spinal cord injury (SCI) are considered to have no chance to recover mobility. Recent breakthrough studies using surgically implanted electrodes over the lumbosacral spinal cord and task-specific training showed some recovery of locomotion in individuals with chronic motor complete SCI. In this study, we used non-invasive continuous electrical stimulation integrated over the cervical, lumbosacral and coccygeal spinal cord combined with targeted alternating activation of flexor and extensor motor pools in severely paralyzed individuals. Non-ambulatory paralyzed individuals who had no prior practice in locomotor training or exposure to spinal stimulation initiated minimally assisted overground stepping immediately when multi-modal stimulation was applied. This breakthrough non-invasive neurorehabilitation technology represents a major advantage for implementation in larger cohorts of individuals and a significant step forward in the rehabilitation of individuals with SCI.

So far, treatment plans for individuals living with clinically complete SCI focus on maintenance therapy above the injury and adaptations to daily activities and life in a wheelchair. Central pattern generation (CPG) discovered in mammals [1] launched over five decades of exploration into whether these mechanisms existed in humans [2]. There is now a preponderance of evidence for the existence of CPG circuitry in humans. This discovery leads to the potential for recovery of locomotion in individuals with clinically complete motor paralysis who historically have little to no chance for mobility. Recent breakthrough studies reported that individuals with chronic motor complete SCI, contrary to their diagnosis and current medical expectation, stood independently, voluntarily generated leg movements, and walked overground with lumbosacral epidural electrical stimulation combined with task-specific motor training [3–5]. These observations showed individuals who were diagnosed to have severely disrupted supraspinal connections to the spinal cord developed new functional connections between the brain and spinal networks in response to epidural stimulation. Concious control was only evident with the combination of epidural stimualtion and task-specific training, and was ultimately required for the observed functional motor benefits. For each study different types of lumbosacral epidural stimulation were used: (1) patterned spatio-temporal alternating stimulation of the flexor-extensor motor pool [6], and (2) continuous stimulation targeting the CPG networks [3–5]. In both approaches, with epidural stimulation and training, positive improvements were observed in stepping with body weight supported walking in incomplete SCI [6] and balance-assisted overground walking in clinically motor complete SCI [4,5]. Further, in a pilot study, the addition of non-invasive cervical transcutaneous stimulation to epidural stimulation of the lumbosacral spinal cord immediately improved the stepping patterns while stepping on a treadmill with body weight support in individuals with motor complete SCI [7].

We present a novel approach of non-invasive multi-modal stimulation that targets multiple spinal cord levels to synergistically activate the neural systems for motor function that also provides evidence of the underlying mechanisms of recovery of human locomotion [8,9] (Fig. 1A, B). This approach combines continuous stimulation to drive the automatic and feed-forward system of CPG networks for stepping pattern generation (T11-T12) with spatio-temporal alternating stimulation targeting dorsal roots (T12-L1; L2-L3) projecting to the leg flexor and extensor motor pools timed to the stepping cycle inducing alternating activity, and continuous cervical spinal cord stimulation (C3-C4) for facilitation of the brain-spinal connectome. Additional sacral-coccygeal (Co1) transcutaneous stimulation further enhances the activation of the human locomotor network [9,10].

**Figure 1.**
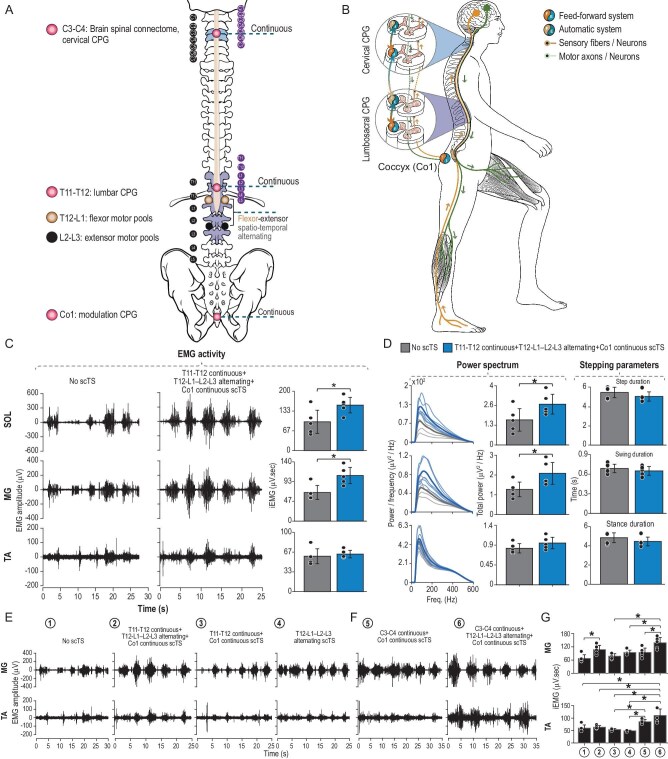
Novel strategy of multi-modal spinal neuromodulation to control stepping movements in individuals with chronic SCI. (A) Schematic representing midline electrode localization for continuous stimulation of the spinal cord at cervical, thoracic, lumbar, and coccygeal (Co1) levels (red circles) as well as lateral placement of the electrodes over the posterior roots for alternating spatial-temporal stimulation to activate flexor (yellow circles) and extensor motor pools (black circles). (B) Shows the interaction of the cervical locomotor network (CPG) and lumbosacral locomotor network (CPG) during stimulation and their modulation by Co1 stimulation. Also shown are the automatic and feed-forward systems at the cervical and lumbosacral levels and interactions between them for the regulation of locomotion. (C) Electromyographic (EMG) activity of leg muscles and EMG characteristics of stepping movements during assisted overground walking without stimulation (no scTS) and independent walking in the presence of continuous and alternating spinal stimulation in participant 1 (AIS A). As can be seen, unlike during the no stimulation condition that demonstrated sporadic and low levels of muscle activity, introducing continuous and alternating stimulation resulted in robust bursting of these muscles during each step cycle. The observed increase was statistically significant* (SOL*: *t* = −5.20, *p* = 0.007; MG*: *t* = −6.11: *p* = 0.004; TA: *t* = −0.929, *p* = 0.405, paired *t*-test). (D) EMG of these leg muscles during trainer-assisted overground locomotion show significant increases in the power spectrum during continuous and alternating stimulation compared to no stimulation. Power spectrum analysis revealed that continuous stimulation and alternating stimulation resulted in a statistically significant* increase in total power (SOL*: *t* = −3.57, *p* = 0.023; MG*: *t* = −3.48, *p* = 0.028; TA: *t* = −2.14, *p* = 0.099, paired *t*-test). More importantly, the other training-related factors that are known to modulate lower limb activity, such as step duration, swing duration and stance duration, did not differ significantly between the two conditions (step duration: *t* = 1.91, *p* = 0.129; swing duration: *t* = 0.81, *p* = 0.462; stance duration: *t* = 1.79, *p* = 0.148, paired *t*-test). (E) These parameters of the locomotor network are most robust in combination compared to when stimulated individually. T11-T12 and Co1 continuous and spatial-temporal stimulation at T12-L1 and L1-L2 eliciting alternating activity timed to the step cycle show they are less robust when stimulated alone. (F) C3-C4 and Co1 continuous stimulation significantly enhances the locomotor activity as shown when combined with alternating stimulation. (G) Bar graph representing significant* increase in muscle activation levels during multi-modal spinal neuromodulation combining continuous and alternating stimulation. Individual dots within the bars represent individual data points (MG: F_(5,24)_ = 8.196, *p* < 0.001; TA: F_(5,24)_ = 13.541, *p* < 0.001, ANOVA followed by *post hoc* Bonferroni correction). Overall, the findings indicate the immediate neuromodulation with integration of the spinal cord networks at various levels results in effective activation of lower limb muscles for locomotion.

This strategy activates related neuronal networks for the initiation and regulation of locomotor function as shown in two naïve chronic SCI individuals who provided written informed consent for these studies (University of Louisville Institutional Review Board ethical approval #19.0434). Participant 1 suffered a complete SCI (AIS A, T9) with zones of partial preservation between T9 and L2 on both sides. Participant 2 suffered an incomplete SCI (AIS C, T9). Both participants presented withspasticity in lower extremity muscles with Modified Ashworth Grading between 1 and 3. Both individuals had no prior practice in locomotor training or exposure to spinal cord neuromodulation, and did not have the ability to perform voluntary overground stepping movements, needing trainers to fully move their legs while stepping without stimulation when attempting to walk overground with balance assistance (Supplementary Video S1 and S2). Then, with the first exposure to multi-modal spinal neuromodulation, there were dramatic changes and trainers only needed to provide minimal assistance because these severely paralyzed individuals could now execute coordinated stepping movements with good postural stability. Representative electromyographic (EMG) activity from the soleus (SOL), medial gastrocnemius (MG) and tibialis anterior (TA) shows that the locomotor pattern dramatically improves during assisted overground stepping immediately upon stimulation when compared to no stimulation (Fig. 1C). The power spectrum of the EMG activity demonstrates an increased neural network contribution with multi-modal stimulation during stepping (Fig. 1D).

The multi-modal stimulation that generated the stepping pattern was a synergistic combination of continuous stimulation at C3-C4, T11-T12 and coccyx spinal levels (30 Hz) and spatio-temporal stimulation of dorsal roots at T12-L1 (30 Hz) for flexor motor pools and L2-L3 (15 Hz) for extensor motor pools. The intensity of stimulation was selected at levels intended to elicit a response while remaining within the comfort range of the participant. The spatio-temporal stimulation was timed to the step cycle using feedback from goniometers at the knee to generate alternating activity in the flexor muscles during the swing phase and extensor muscles during the stance phase (Fig. 1E). Enhancement of the locomotor pattern is observed when coupling C3-C4 stimulation (30 Hz) with alternating L2-L3 stimulation [9]. This electrical stimulation above the site of injury at the cervical level regulates the brain-spinal connectome, reactivating descending dormant systems and driving mechanisms that improve interlimb coordination [7]. This aligns with multi-modal scTS applied simultaneously to the cervical, thoracic, and lumbar spinal cord facilitating stepping performance in non-injured individuals more effectively than when compared to stimulation at T11-T12 only [10]. These results support the computational modeling of current field distribution simulations, that non-invasive multi-modal stimulation of the spinal cord at different sites can stimulate distinct neural structures and can serve to link the stimulation sites to targeted spinal structures [9].

We hypothesized that when merged, these inputs can synergistically regulate the locomotor networks to facilitate recovery of stepping through a multi-system integrative approach. Both traditional and modern neurorehabilitation strategies targeting motor functions are based on the neuroplasticity concept, suggesting that a large number of task-specific motor repetitions are needed to provide structure and functional reorganization of the spinal cord to recover stepping movements. It was reported that only after a hundred locomotor training sessions with lumbosacral epidural stimulation, the SCI individuals (AIS A) recovered the ability to independently walk on the treadmill with body weight support and external balance control [5]. We report that this level of recovery can occur immediately when synergistic multi-modal non-invasive spinal stimulation is strategically used. We observed the initiation of the stepping movements in SCI individuals instantly when continuous and spatio-temporal alternating spinal stimulation was simultaneously applied at multiple stimulation sites.

This approach may be more feasible for large-scale translation to the SCI community due to lower costs and no requirement for surgery. Education and training are needed because the success of independent stepping movements relied on selecting the specific site of stimulation with accurate electrode placement and stimulation parameters in order to elicit the desired response. For use in the home and community, carry-over improvements not needing stimulation would be needed or further advancements in technology required for devices to be able to be used outside of the clinic.

For the first time, neuromodulation offers a viable treatment for motor recovery moving those living with chronic SCI beyond compensation and maintenance therapies. The benefits of improved quality of life, community participation, and long-term lower medical costs are immense and now are within reasonable reach. The benefit of epidural stimulation in the recovery of motor function in chronic SCI has compelling evidence, surgical implantation is required with the targets for neuromodulation limited to the paddle coverage. The strategy of synergistic multi-modal non-invasive activation of locomotor systems localized in different parts of the spinal cord demonstrates the integration of critical spinal mechanisms for effective regulation of locomotion and may become a breakthrough neurorehabilitation technology.

## Supplementary Material

nwaf239_Supplemental_Files

## DATA AND MATERIALS AVAILABILITY

All data, code and materials used in the analysis will be made available on reasonable request to the authors.

## References

[1] Grillner S . Science 1985; 228: 143–9. 10.1126/science.3975635 3975635

[2] Dimitrijevic MR, Gerasimenko Y, Pinter MM. Ann NY Acad Sci 1998; 860: 360–76. 10.1111/j.1749-6632.1998.tb09062.x 9928325

[3] Harkema S, Gerasimenko Y, Hodes J et al. The Lancet 2011; 377: 1938–47. 10.1016/S0140-6736(11)60547-3 PMC3154251 21601270

[4] Angeli CA, Boakye M, Morton RA et al. N Engl J Med 2018; 379: 1244–50. 10.1056/NEJMoa1803588 30247091

[5] Gill ML, Grahn PJ, Calvert JS et al. Nat Med 2018; 24: 1677–82. 10.1038/s41591-018-0175-7 30250140

[6] Wagner FB, Mignardot J-B, Le Goff-Mignardot CG et al. Nature 2018; 563: 65–71. 10.1038/s41586-018-0649-2 30382197

[7] Angeli CA, Gerasimenko Y. Front Bioeng Biotechnol 2023; 11: 107316. 10.3389/fbioe.2023.1073716 PMC9932494 36815892

[8] Moshonkina T, Grishin A, Bogacheva I et al. Frontiers in Human Neuroscience 2021; 14: 622533. 10.3389/fnhum.2020.622533 33519405 PMC7838433

[9] Siu R, Brown EH, Mesbah S *et el*. J Clin Med 2022; 11: 3670. 10.3390/jcm11133670 35806954 PMC9267673

[10] Gerasimenko YP, Lu DC, Modaber M et al. J Neurotrauma 2015; 32**:** 1968. 10.1089/neu.2015.4008 26077679 PMC4677519

